# Biological Mechanisms in Pregnant Women With Anxiety (Happy Mother-Healthy Baby Supplement Study): Protocol for a Longitudinal Mixed Methods Observational Study

**DOI:** 10.2196/43193

**Published:** 2023-04-11

**Authors:** Morgan L Sherer, Abid Malik, Lauren M Osborne, Armaan A Rowther, Ahmed Zaidi, Najia Atif, Atif Rahman, Lubna E Kahloon, Muhammad Salman, Gayane Yenokyan, Pamela J Surkan

**Affiliations:** 1 Johns Hopkins Center for Women’s Reproductive Mental Health Departments of Psychiatry & Behavioral Sciences and Gynecology & Obstetrics Johns Hopkins School of Medicine Baltimore, MD United States; 2 Human Development Research Foundation Gujar Khan Pakistan; 3 Department of International Health Johns Hopkins Bloomberg School of Public Health Baltimore, MD United States; 4 Department of Psychological Sciences University of Liverpool Liverpool United Kingdom; 5 Department of Obstetrics and Gynecology Holy Family Hospital Rawalpindi Medical University Rawalpindi Pakistan; 6 Public Health Laboratory Division National Institute of Health Islamabad Pakistan; 7 Johns Hopkins Biostatistics Center Department of Biostatistics Johns Hopkins Bloomberg School of Public Health Baltimore, MD United States

**Keywords:** antenatal anxiety, perinatal anxiety, pregnancy, cognitive behavioral therapy, inflammation, allopregnanolone, Pakistan, randomized controlled trial

## Abstract

**Background:**

Anxiety and depression are common in the perinatal period and negatively affect the health of the mother and baby. Our group has developed “Happy Mother-Healthy Baby” (HMHB), a cognitive behavioral therapy–based psychosocial intervention to address risk factors specific to anxiety during pregnancy in low- and middle-income countries (LMICs).

**Objective:**

The purpose of this study is to examine biological mechanisms that may be linked to perinatal anxiety in conjunction with a randomized controlled trial of HMHB in Pakistan.

**Methods:**

We are recruiting 120 pregnant women from the Holy Family Hospital, a public facility in Rawalpindi, Pakistan. Participants are assessed for at least mild anxiety symptoms using the Hospital Anxiety and Depression Scale (ie, a score ≥8 on the anxiety scale is necessary for inclusion in the anxiety groups and <8 for inclusion in the healthy control group). Women who meet the criteria for an anxiety group are randomized into either the HMHB intervention group or an enhanced usual care (EUC) control group. Participants receive HMHB or EUC throughout pregnancy and undergo blood draws at 4 time points (baseline, second trimester, third trimester, and 6 weeks post partum). We will assess peripheral cytokine concentrations using a multiplex assay and hormone concentrations using gas chromatography and mass spectrometry. The statistical analysis will use generalized linear models and mixed effects models to assess the relationships across time among anxiety, immune dysregulation, and hormone levels, and to assess whether these biological factors mediate the relationship between anxiety and birth and child development outcomes.

**Results:**

Recruitment started on October 20, 2020, and data collection was completed on August 31, 2022. The start date for recruitment for this biological supplement study was delayed by approximately half a year due to the COVID-19 pandemic. The trial was registered at ClinicalTrials.gov (NCT03880032) on September 22, 2020. The last blood samples were shipped to the United States on September 24, 2022, where they will be processed for analysis.

**Conclusions:**

This study is an important addition to the HMHB randomized controlled trial of an intervention for antenatal anxiety. The intervention itself makes use of nonspecialist providers and, if effective, will represent an important new tool for the treatment of antenatal anxiety in LMICs. Our biological substudy is one of the first attempts to link biological mechanisms to antenatal anxiety in an LMIC in the context of a psychosocial intervention, and our findings have the potential to significantly advance our knowledge of the biological pathways of perinatal mental illness and treatment efficacy.

**Trial Registration:**

ClinicalTrials.gov NCT03880032; https://clinicaltrials.gov/ct2/show/NCT03880032

**International Registered Report Identifier (IRRID):**

DERR1-10.2196/43193

## Introduction

Common perinatal mental disorders such as depression and anxiety occur frequently, are underdiagnosed, and negatively affect the health of both the mother and baby [1]. While the majority of epidemiological data is focused on perinatal depression, peripartum anxiety is equally prevalent, if not more so, yet remains largely understudied [2]. In Pakistan, for example, between one-third and one-half of pregnant women experience anxiety during pregnancy [3,4]. Despite the known morbidity associated with antenatal anxiety (ie, high risk of later anxiety or depression, risk of suicide [5-8], a range of poor birth outcomes [9-11], ill child health [12], and host of child development problems [13-17]), the biological underpinnings of antenatal anxiety remain unknown. In low- and middle-income countries (LMICs), the treatment gap for anxiety disorders exceeds 75%, largely due to the absence of specialists to provide evidence-based psychological interventions [18]. To address this gap, our group developed a cognitive behavioral therapy–based psychosocial intervention called “Happy Mother-Healthy Baby” (HMHB), delivered by nonspecialists and tailored to address risk factors specific to anxiety during pregnancy. HMHB is the subject of an ongoing randomized controlled trial in Rawalpindi District, Pakistan [19,20]. In conjunction with this larger study, our group has designed an observational study to examine biological factors that may be linked to perinatal anxiety, with a particular focus on immune dysregulation and sex hormones. Any such alterations can inform plausible mechanisms that can then be used in the development of other interventions, either psychosocial or biological (for example, anti-inflammatory nutritional approaches or exercise interventions).

The connection between anxiety and immune dysregulation outside of pregnancy is well-established [21,22], including studies showing heightened anxiety symptoms in women with inflammatory diseases such as colorectal cancer and irritable bowel syndrome [23-25]. There are several mechanisms by which increased inflammation could be related to fear and anxiety. For example, exposure to stressful stimuli has been shown to activate immune cells to release cytokines and other peripheral inflammatory markers [26-29]. This increased inflammation can be related to hypothalamic-pituitary-adrenal axis dysregulation and, in turn, to behavioral manifestations and clinical symptoms of anxiety [26-32]. Such changes may be especially important in pregnancy, as maternal immune activation has been implicated as a risk factor for child developmental outcomes such as altered neurobehavioral functioning [33,34]. Even a healthy pregnancy is a time of immune dysregulation [35-37]. Prior research has identified a distinct “cytokine fingerprint” in normal pregnancy—a pattern of relative changes in cytokine levels across time, despite individual differences in absolute levels—due in large part to hormonally driven decreases in both innate and adaptive immunity [37-40].

**Figure 1:**
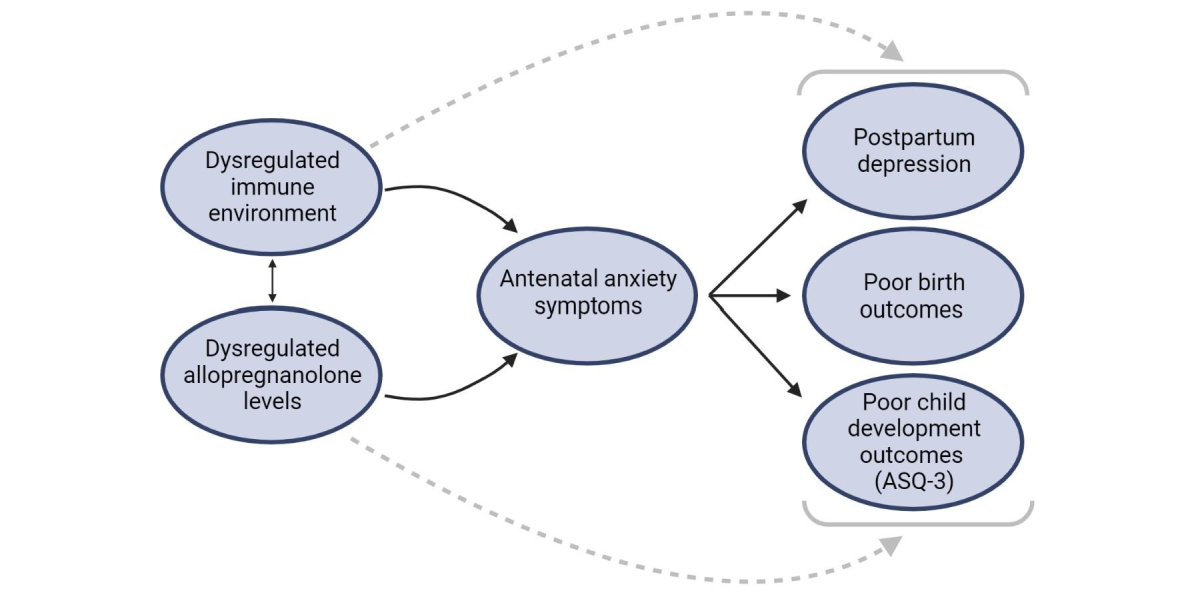
Proposed theoretical model. In our study, we will measure the relationship between the dysregulated immune and endocrine environment, their influence on antenatal anxiety symptoms, and the direct and indirect influence on associated outcomes (postpartum depression, poor birth outcomes, and poor child development outcomes). ASQ-3: Ages and Stages Questionnaire.

The neuroendocrine system is centrally related to the immune response in pregnancy. Progesterone (P4) and its metabolites, including allopregnanolone (ALLO), rise dramatically in healthy pregnancy [41], and there is bidirectional feedback between P4 metabolites and the immune system [42-46]. Higher levels of ALLO have been linked to decreased production of inflammatory mediators by microglia, reduced cytokine production by macrophages, and diminished lymphocyte proliferation [47]. Low levels of ALLO have also been linked to anxiety outside of pregnancy [48]. There is some evidence of a relationship between ALLO and psychiatric symptoms in the perinatal period as well [2,49-51]. A theoretical model outlining these possible connections is given in Figure 1.

The proposed study aims to investigate the biological mechanisms that underlie antenatal anxiety in women enrolled in the HMHB psychological intervention in Pakistan. This study sets out to answer the following research questions: (1) whether there is a pattern of immune dysregulation common to women with antenatal anxiety, (2) whether there is a relationship between ALLO and concurrent anxiety symptoms or future symptoms of postpartum depression, (3) whether immune dysregulation is related to dysregulation of ALLO, (4) whether these biological mechanisms are mediators of the association between antenatal anxiety and poor birth outcomes, and (5) if biological markers of anxiety are associated with infant neurodevelopment at 6 weeks of age as measured by the Ages and Stages Questionnaire (ASQ-3).

While the evidence concerning the role of such biological factors is increasing, no study has yet examined them in the context of a psychological intervention in pregnancy in an LMIC, where anxiety and depression rates are high and psychosocial intervention can make a meaningful difference [52]. This manuscript describes the design and implementation of one such study.

## Methods

### Study Design and Setting

Pregnant women are recruited from the Holy Family Hospital (HFH), a public facility in Rawalpindi, Pakistan.

### Recruitment

The study is embedded in the main trial of the HMHB intervention, for which the recruitment of 1200 women started in April 2019 [20]. Recruitment for this supplemental biological study began in October 2020. Recruitment is carried out by a female assessment team based in the outpatient Gynecology and Obstetrics Department. The team describes the HMHB study to all pregnant women who fulfill the initial screening criteria (see next paragraph for details on level 1 screening) and their families visiting the department for their initial prenatal visit at or before 22 weeks’ gestation (approximately 600 women monthly). For this biological supplement study, women are included if they are ≤12 weeks of gestation. All women eligible for the main study are offered the opportunity to participate in the supplemental study, and we anticipate enrollment of 120 participants over 18 months (30 from each arm of the main study and another 60 pregnant controls who are neither depressed nor anxious as assessed by the Hospital Anxiety and Depression Scale [HADS]); this rate of recruitment is slower than for the main study, due to the reluctance of many women to undergo blood draws, as well as stricter inclusion criteria related to gestational age and absence of anemia.

### Screening Procedure and Eligibility Criteria

We use 3 levels of inclusion and exclusion screening criteria that occur at the same time at enrollment. For level 1 screening, we determine if the woman is at ≤12 weeks’ gestation, ≥18 years old, resides ≤20 km from HFH, and understands Urdu. If they fulfill these criteria, women expressing initial interest are asked to provide informed consent for completing the next stage of screening. At level 2 screening, we determine from self-reports if women have (1) life-threatening health conditions, including suicidal ideation, (2) past or current serious learning disability, (3) past or current psychiatric disorder other than anxiety (eg, bipolar disorder or schizophrenia) or psychiatric treatment (eg, current use of anxiolytic drugs or other psychotropic drugs), (4) medical disorders or severe maternal morbidity that require current inpatient management, or (5) intensive care unit admission (for treatment, not solely for assessment). If not excluded on this basis, potential participants undergo level 3 screening, which includes assessment for the presence or absence of at least mild anxiety on the anxiety subscale of the HADS (ie, a score ≥8 is necessary for inclusion in the anxiety groups and <8 on both the anxiety and depression subscales for the healthy controls). The HADS is a validated screening tool for depression and anxiety and has been used in research settings [53]. Women who score ≥8 on the HADS anxiety subscale move onto level 4 screening, which includes an interview with a qualified psychiatrist who conducts a Structured Clinical Interview for DSM V Diagnoses; women meeting criteria for a major depressive episode are excluded.

### Randomization

Participants with anxiety are randomly assigned to a group using a pseudo-random number generator, which assigns a random sequence via permuted blocks of sizes 4, 8, 12, and 16. The list of assignments is printed out, and each step in the sequence is stored apart from the others in an opaque envelope; the envelopes are numbered sequentially with a 7-digit code. Participants are assigned to control or intervention according to the next available envelope after consent. All members of the on-site trial team, as well as the trial statistician, principal investigator, and coinvestigator leading the biological study, are blinded to the allocation.

### Participant Groups

To enhance participation in the study and adherence to study protocols, all participants are reimbursed for transportation and for as many ultrasounds as are medically indicated at HFH during pregnancy.

#### Intervention Group

Women allocated to this group receive the HMHB intervention, a cognitive behavioral therapy–based treatment focused on anxiety and culturally adapted and tailored to a low-literacy population during the initial research phase [19,54]. Core principles include the mother’s psychosocial well-being, preparation for birth, relationship skills, and psychosocial support [19]. HMHB includes 6 core weekly sessions and anywhere from 2 to 6 booster sessions, depending on the timing of enrollment and individual needs. The final core session is in the third trimester of pregnancy. HMHB is presented as a “mother-to-be” program (to combat the stigma associated with mental health issues), and family members are encouraged to participate in 3 sessions. The intervention is delivered by nonspecialist research therapists, who are university graduates with a master’s degree in psychology but no experience delivering mental health care [55]. Prior to the start of the study, research therapists receive a 6-day classroom training, followed by field training and weekly group supervision. Competency is assessed by role plays during training and by direct observation of 15% of their sessions by independent assessors using the ENACT (Enhancing Assessment of Common Therapeutic Factors) rating scale for global training and supervision in mental health [56].

#### Enhanced Usual Care (EUC) Control Group

Women with anxiety allocated to the control condition are receiving EUC in the Obstetrics Department. The target number of visits is 8, as recommended by the World Health Organization (WHO) for a positive pregnancy experience [57]. Visits involve evaluating health status, discussing any concerns, and performing routine exams consistent with the stage of pregnancy.

#### Healthy Controls

The healthy control group consists of 60 women who have low or no anxiety or depressive symptoms (<8 on both the anxiety and depression subsections of the HADS). This will allow us to compare biological processes between women with anxiety (both those receiving the intervention and those in the EUC) and healthy controls.

### Timing of and Measures for Data Collection

**Figure 2:**
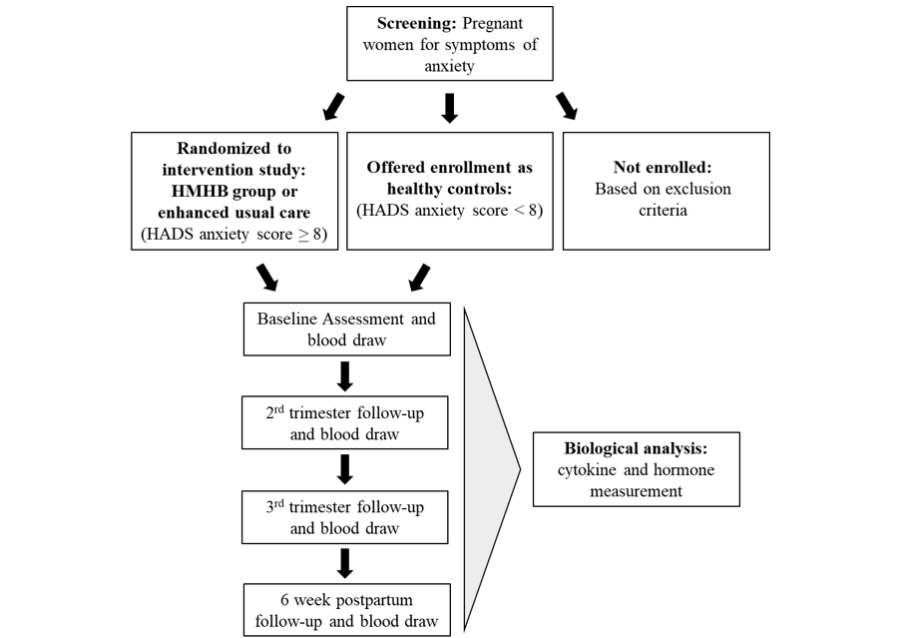
Study design and setting. HADS: Hospital Anxiety and Depression Scale; HMHB: Happy Mother-Healthy Baby.

All participants in the main study with anxiety (either in the intervention group or receiving EUC) will undergo the same visits and psychological measures. The measures received by the healthy controls are indicated in the final column of Table 1. The timelines of visits and measures used are outlined in Table 1 and Figure 2, and details of these measures are described in the trial protocol paper for the main study [20].

**Table 1:** Timing of data collection.

			Measured in anxious women (intervention and anxious controls)											Measured in healthy controls
			Screening or baseline^a^ (time 1)		Second trimester (time 2)		Third trimester (time 3)		Birth (time 4)		6 weeks post partum (time 5)		Time-matched	
**Assessments**														
	Sociodemographic questions	✓										✓		
	PSS-10^b^ (stress)					✓				✓				
	PES-Brief^c^ (pregnancy anxiety)	✓				✓								
	PHQ-9^d^ (depressive symptoms)									✓				
	HADS^e^ depression (subscale)	✓								✓		✓		
	HADS anxiety (subscale)	✓		✓		✓				✓		✓		
	MDE^f^ diagnosis (from SCID^g^)	✓								✓				
	MSPSS^h^ (social support)	✓				✓				✓				
	MRQ^i^ (relationships)	✓				✓				✓				
	Empowerment	✓								✓				
	Pakistan Demographic Health Survey interpersonal violence questions									✓				
	Breastfeeding									✓		✓		
	PBQ^j^ (bonding)									✓				
	MIRI^k^ (maternal responsiveness)									✓				
	MSEQ^l^ (maternal self-efficacy)									✓				
	Blood draw for biological factors	✓		✓		✓				✓		✓		
	ASQ-3^m^									✓		✓		
**Primarily from medical records**														
	Pregnancy conditions					✓						✓		
	Birth outcomes: SGA^n^, PTB^o^, LBW^p^							✓				✓		
	Medications	✓				✓				✓		✓		

^a^Baseline assessment may be the same day or up to 2 weeks after screening.

^b^PSS-10: Perceived Stress Scale 10 items.

^c^PES-Brief: Pregnancy Experience Scale-Brief version.

^d^PHQ-9: Patient Health Questionnaire 9 items.

^e^HADS: Hospital Anxiety and Depression Scale.

^f^MDE: Major Depressive Episode.

^g^SCID: Structured Clinical Interview for DSM Disorders.

^h^MSPSS: Multidimensional Scale of Perceived Social Support.

^i^MRQ: Motivations for Reading Questionnaire.

^j^PBQ: Postpartum Bonding Questionnaire.

^k^MIRI: Maternal Infant Responsiveness Instrument.

^l^MSEQ: Maternal Self-Efficacy Questionnaire.

^m^ASQ-3: Ages and Stages Questionnaire.

^n^SGA: small for gestational age.

^o^PTB: preterm birth.

^p^LBW: low birth weight.

### Biological Sample Collection

Participants have their blood drawn (up to 20 ml) at 4 visits by a phlebotomist who was trained on the study protocol. Target times for sampling blood are at baseline (between 10 and 22 weeks’ gestation), during the second trimester (between 22 and 26 weeks’ gestation), during the third trimester (between 34 and 38 weeks’ gestation), and between 4 and 8 weeks post partum. Each participant is scheduled as close as possible to the same time and during morning hours for all 4 blood draws (as blood is drawn in HFH between 8 AM and 1 PM), allowing us to account for diurnal variation in cytokine levels. Blood is centrifuged within 8 hours of collection using low-density gradient centrifugation via a Z326 High Performance Centrifuge. Plasma is immediately frozen in cryovials in a ThermoScientific Revco RLE Ultra-Low Lab Freezer at –80 °C and stored at the National Institute of Health Laboratory in Pakistan until it is shipped to Baltimore.

### Shipping of Samples

Plasma samples (approximately 1280 cryovials) are being shipped from Pakistan to Baltimore, United States, in multiple shipments throughout the trial using courier services. Blood is kept frozen at a maximum temperature of –20 °C throughout shipment using standard cold packs, dry ice, or liquid nitrogen as required by local regulations and the standards of the courier services. All plasma samples are then stored at –80 °C in a ThermoScientific Revco RLE Ultra-Low Lab Freezer at Johns Hopkins to await analysis together at the end of the study to avoid batch effects.

### Analysis of Biological Samples

#### Cytokine Analysis

Plasma cytokines will be measured using the Meso Scale Discovery (Gaithersburg, MD) Ultrasensitive Pro-inflammatory multiplex kit. The Meso Scale Discovery multispot array will be run according to the manufacturer's protocol. Calibration curves will be prepared. Plates will be read using the MS2400 imager. Samples for cases and controls will be assayed adjacently but in random order and replicated; the technician performing analyses will be unaware of the case status. To determine assay reliability, we will calculate the coefficient of variation for each woman's replicates when both have concentrations above the limit of detection [58].

#### Hormone Analysis

All hormone analyses will take place at the same time at the end of the study. Samples will be stored at –80 °C and run in 1 batch. Extraction, derivatization, and gas chromatography and mass spectrometry analyses of ALLO will be performed as previously described [59,60]. After the samples are extracted with ethyl acetate and lyophilized, the steroids of interest will be purified and separated by high-performance liquid chromatography. Tritiated neuroactive steroids will be added to monitor the high-performance liquid chromatography retention profile, while deuterated internal standards consisting of 2 pmol of deuterium-labeled neuroactive steroids will be used to allow quantitation of the compound of interest and correct for procedural losses. Each steroid of interest will then be derivatized for gas chromatography and mass spectrometry [59,60]; mass spectrometry analysis will be performed in the standard electron impact mode for both ALLO and its isomers and P4 measurements. The quantity of each neuroactive steroid of interest will be calculated by dividing the area under the peak of the neuroactive steroid in the sample by the area under the peak of the deuterated internal standard.

### Statistical Analysis

#### Aim 1: Immune Dysregulation and Anxiety

##### Models for Immune Marker Patterns

Initially, we will perform exploratory cross-sectional analyses at each time point, both for quality assurance and to attain a better understanding of the distributions of the outcome variables for use in subsequent analyses (eg, the need for any transformations), followed by the longitudinal analyses. Specifically, the distribution of cytokine values will be explored at each time point to assess the frequency of missingness (including the number of observations below the limit of detection) and summarized using descriptive statistics. Longitudinal models will be fit to the cytokine data to describe the average trajectory over the course of pregnancy. Generalized linear mixed-effects models (GLMMs) will be fit with random intercepts for women to account for within-woman correlation of the measurements over time. The basic model will include time, group (intervention, EUC, and healthy control), and their interaction, and will address whether cytokine trajectories differ between groups. This basic model will be extended to include a random slope for time if the exploratory analysis shows large variability in individual woman-specific cytokine trajectories over time. Additionally, nonindependent residual correlation structures will be explored to assess any leftover correlation after accounting for the random-effect structure. The main model will include log-transformed cytokine values; however, other distributions for the dependent variable will be used, such as multilevel Tobit models that account for the lower limit of detection for these measurements. GLMMs account for “missing at random” missingness, that is, women with missing data at some time points are not excluded, and missing cytokine values are assumed to depend on observed data: prior values and covariates in the model [61]. These models allow us to estimate each woman’s trajectory as a deviation from the average trend using all their available data by borrowing information within and between women. However, in the case of substantial dropout, we will explore pattern mixture models where the “missing at random” assumption might not be adequate. These models allow us to estimate model coefficients for each pattern of drop-out or missing data and obtain overall estimates by averaging over the patterns [62]. Standard model checking will be performed, and the most parsimonious model will be selected based on the Bayesian information criterion.

##### Immune Dysregulation and Anxiety

To characterize the relationship between anxiety symptoms and peripheral markers of inflammation, we will assess the associations between cytokine levels as independent variables and the level of anxiety (as measured by the HADS-anxiety) as the dependent variable. The level of anxiety will be considered from the following perspectives: (1) dichotomous (HADS-anxiety score ≥8 or <8) and (2) continuous. In both analyses, we will compare women with anxiety against healthy controls to evaluate whether there is a positive association between cytokines and anxiety at different time points during pregnancy. In the exploratory analyses, we will also compare the distribution of demographic, medical, and pregnancy history characteristics across the 3 study groups to assess for potential confounding.

We will add anxiety measured as an independent variable to the model identified in the first set of analyses for cytokines as a dependent variable. The models will adjust for baseline symptom severity and other patient characteristics that are differentially distributed across the 3 study arms.

#### Aim 2: ALLO and Anxiety

To determine the relationship between levels of ALLO in pregnancy and anxiety symptoms, we will follow the same approach as for longitudinal modeling immune dysregulation, with ALLO levels as the independent variable and anxiety as the dependent variable. The model will include group and time as well as interactions of ALLO with group (to test whether the relationship between ALLO and anxiety differs by group) and ALLO with time (to test whether the relationship between ALLO and anxiety differs by time). The model will use concurrent values and lagged values for ALLO and anxiety to test whether the ALLO levels at prior time points are associated with the current value of anxiety scores.

Finally, because anxiety in pregnancy is a major risk factor for postpartum depression and our previous work has shown a relationship between ALLO in pregnancy and future postpartum depression [2,51], we will repeat all analyses using the development of postpartum depression as the dependent variable.

#### Aim 3: Immune Dysregulation and ALLO

We will evaluate the associations between ALLO levels and immune markers across time during pregnancy by disease group using exploratory analytic techniques, including visual displays to inform modeling. Using a similar GLMM framework, we will fit a separate model for each immune marker with ALLO levels as the independent variable, in addition to group, time, and other characteristics identified as potential confounders. Cytokine-specific models can be combined in 2 ways: (1) by creating a composite cytokine score for each woman, and (2) by fitting a multilevel model with crossed random effects that include random intercepts for women and immune markers to “borrow information” across multiple immune markers within the woman.

#### Aim 4: Mediation Analysis by Biological Measures

Estimated anxiety trajectories and ALLO levels (ie, random slope estimates from the GLMMs) will be used as candidate independent variables in generalized linear models (GLMs) for risk of pregnancy and birth outcomes: pre-eclampsia, gestational diabetes, preterm birth (PTB), small for gestational age (SGA), and low birth weight (LBW). GLM with a Poisson distribution will be used to model risk for rare outcomes. The cross-validated area under the curve will be used to assess model discrimination and determine which model is the best fit for our data.

The next analysis will focus on the association of antenatal anxiety and birth outcomes, comparing anxious to nonanxious women. We will specifically assess mediation by biological factors in both cross-sectional and longitudinal analyses, partitioning the total effect of anxiety on birth outcomes as direct and indirect, through a change in cytokine and hormone levels.

#### Aim 5: Infant Neurodevelopment

The final analysis will examine the association between biological indicators of anxiety and infant neurodevelopment at 6 weeks post partum using the ASQ-3, comparing both groups of women with symptoms of anxiety (those who received the intervention and those who did not) to the healthy controls and controlling for ALLO and immune dysregulation. The same type of GLM described in the previous section will be used with appropriate distributions, such as Gaussian for continuous or normally distributed outcomes or Poisson for binary outcomes. We will also examine whether the severity of anxiety symptoms modifies the effect. Because the ASQ-3 can be used as both a dichotomous and continuous scale, we will examine it using both binary and continuous techniques.

#### Sample Size and Power

A sample size of 60 participants in each group, women with anxiety and healthy controls, will give us at least 80% power to detect a difference in the means of log-transformed cytokines across groups with an effect size of 0.3 to 0.5. This calculation assumes 4 repeated measurements with an exchangeable covariance structure, a correlation of 0.6, and an =.05. Sample size calculations were performed using the PASS Sample Size Software program (NCSS Statistical Software) [63]. Prior studies in our clinic in Baltimore have an attrition rate of up to 20%, but the attrition rate for this type of study in Pakistan is unknown.

### Ethics Approval

#### Institutional Approval

This study was reviewed and approved by the Johns Hopkins Bloomberg School of Health Institutional Review Board, Baltimore, United States (IRB No. 9177/MOD1879), the Human Development Research Foundation Ethics Committee, Islamabad, Pakistan (IRB/001/2017), the RMU Institutional Research Forum, Rawalpindi, Pakistan (IRB/RMU-20/12/20190), the National Bioethics Committee of Pakistan, Islamabad, Pakistan (IRB No.4-87/NBC-536/20/881), and the National Institute of Mental Health (NIMH)–appointed Global Mental Health Data Safety and Monitoring Board from the United States.

#### Consent Procedures

Following WHO guidelines [64], full study information is provided in Urdu before any study participant is asked to provide written informed consent. In cases of illiteracy, information is read aloud in the presence of a witness (not affiliated with HMHB), and oral consent is witnessed. These procedures as alternatives to written informed consent are routine and culturally acceptable in Pakistan.

#### Care and Referrals

Detailed ethical procedures have been established for the main trial. These procedures are the same for the extra 60 healthy (nonanxious) controls added for this substudy. Any woman showing severe depression or suicidality at any point during the study will be referred directly for psychiatric or psychological care. For non–life-threatening but high-symptom levels of anxiety or depression, the obstetrician or primary care provider will be alerted to undertake further assessment and to make appropriate referrals. Based on any report of intimate partner violence (IPV), during assessments, or reports of IPV in answer to a screener asked (at baseline and the third trimester) or on questions about emotional and physical violence taken from the Pakistan Demographic Health Surveys asked at 6 weeks post partum, women who report IPV will be referred to the Institute of Psychiatry, Rawalpindi Medical College, for social services.

### Dissemination

The HMHB intervention is designed to reduce symptoms of anxiety during pregnancy, which has the potential to affect the development of common maternal postpartum perinatal mental disorders, including postnatal depression and anxiety, as well as intrauterine growth, as reflected in SGA, LBW, and PTB. The findings from this study will describe the biological mechanisms through which antenatal anxiety may be linked to future maternal psychiatric risk as well as how these biological markers may relate to birth and child development outcomes. The principal investigator and coinvestigators, both in the United States and the host country, will be given access to the cleaned data sets. The deidentified project data from the study will be posted on ClinicalTrials.gov and the NIMH data archive. The study results will be disseminated through peer-reviewed journals and conferences.

## Results

Recruitment started on October 20, 2020, and data collection was completed on August 31, 2022. The start date for recruitment for this biological supplement study was delayed by approximately half a year due to the COVID-19 pandemic. The trial was registered at ClinicalTrials.gov (NCT03880032) on September 22, 2020. The last shipment of blood samples was shipped to the United States on September 24, 2022, where they will be processed for analysis. At the time of this submission on November 27, 2022, data had been collected but biological and data analysis had not yet begun.

## Discussion

This study is an important addition to the HMHB randomized controlled trial of an intervention for antenatal anxiety. The intervention itself makes use of nonspecialist providers and, if effective, will represent an important new tool for the treatment of antenatal anxiety in LMICs. Our biological substudy is one of the first attempts to link biological mechanisms to antenatal anxiety in an LMIC in the context of a psychosocial intervention, and our findings have the potential to significantly advance our knowledge of the biological pathways of perinatal mental illness and treatment efficacy.

### Expected Principal Findings

We hypothesize that anxious women will exhibit increased innate immune activity in late pregnancy compared to healthy controls. We further hypothesize that pregnancy ALLO levels will be negatively correlated with anxiety and that low ALLO in early- and midpregnancy will predict increased immune activation in late pregnancy. Lastly, we hypothesize that adverse birth outcomes will be positively correlated with immune activation and negatively correlated with pregnancy ALLO and that the neurobehavioral functioning of babies born to women with anxiety will be correlated with biological indicators when compared to infants born to healthy controls.

### Comparison to Prior Work

These hypotheses are centered on our previous work finding elevations of innate immune cytokines in women with anxiety during pregnancy and early postpartum and our work finding that low ALLO in pregnancy is associated with current and future symptoms of anxiety [1,2,65]. These prior studies were carried out in extremely different populations (both in a high-income country, one with largely low-income Black and Hispanic participants and the other with largely high-income White participants). Should we find alterations in immune and/or neurosteroid activity, we will advance the field’s understanding of the biological mechanisms that may underlie perinatal mood and anxiety disorders and pave the way for future research into new methods of treatment to address these potential mechanisms.

### Strengths and Limitations

Our study involves research teams on 3 continents and the training of a substantial number of nonspecialist providers. It is complex to begin with. Nevertheless, recruitment for both the main study and the supplement study has been steady. The biggest logistical challenge has been the COVID-19 pandemic (combined in Pakistan with waves of dengue fever). These public health crises delayed the start of the study due to the lockdown and have greatly reduced our ability to recruit during the rest of the study period. Pregnant women in Pakistan have been reluctant to come into the hospital even for standard prenatal care due to the pandemic, and there have been misconceptions and misinformation about the role of public hospitals in COVID-19 [66]. These logistical challenges have resulted in a scaling back of our study design. We had originally planned to recruit larger numbers (N=300) into this substudy, which would have allowed us to examine not only differences between anxious and healthy women but also whether those differences were mitigated by our psychosocial intervention. These latter questions will now await a follow-up study.

### Future Directions

Our next step for this research is to analyze the biological samples collected. Future studies may include additional secondary biological analyses and the design of future studies involving other maternal and infant biological systems.

### Trial Status

This article reports on the first version of our protocol; recruitment began in October 2020 and ended in January 2022; study visits ended on August 31, 2022.

## Appendix

Multimedia Appendix 1 Peer-reviewer report from Biobehavioral Mechanisms of Emotion, Stress and Health (MESH) Study Section - Biobehavioral and Behavioral Processes Integrated Review Group - Center for Scientific Review (National Institutes of Health, USA).

## Abbreviations

ALLO: allopregnanolone
ASQ-3: Ages and Stages Questionnaire
EUC: enhanced usual care
GLM: generalized linear model
GLMM: generalized linear mixed-effects models
HADS: Hospital Anxiety and Depression Scale
HFH: Holy Family Hospital
HMHB: Happy Mother-Healthy Baby
IPV: intimate partner violence
LBW: low birth weight
LMIC: low- and middle-income country
NIMH: National Institute of Mental Health
P4: progesterone
PTB: preterm birth
SGA: small for gestational age
WHO: World Health Organization

## Data Availability

The HMHB intervention is designed to reduce symptoms of anxiety during pregnancy, which has the potential to affect the development of common maternal postpartum perinatal mental disorders, including postnatal depression and anxiety, as well as intrauterine growth, as reflected in SGA, LBW, and PTB. The findings from this study will describe the biological mechanisms through which antenatal anxiety may be linked to future maternal psychiatric risk as well as how these biological markers may relate to birth and child development outcomes. The principal investigator and coinvestigators, both in the United States and the host country, will be given access to the cleaned data sets. The deidentified project data from the study will be posted on ClinicalTrials.gov and the NIMH data archive. The study results will be disseminated through peer-reviewed journals and conferences.
