# Supplementary material for: Biological Mechanisms in Pregnant Women With Anxiety (Happy Mother-Healthy Baby Supplement Study): Protocol for a Longitudinal Mixed Methods Observational Study
Source: JMIR Res Protoc. 2023 Apr 11;12:e43193. doi: 10.2196/43193 (PMC10132042; doi:10.2196/43193)
Supplement: Multimedia Appendix 1 [file resprot_v12i1e43193_app1.pdf]

SUMMARY STATEMENT

(Privileged Communication)

PROGRAM CONTACT:
Makeda Williams
301-435-4582
willimak@mail.nih.gov

Release Date: 03/08/2019
Revised Date:

Application Number: 3 R01 MH111859-03S1

Principal Investigator

SURKAN, PAMELA J

Applicant Organization: JOHNS HOPKINS UNIVERSITY

Review Group: MESH
Biobehavioral Mechanisms of Emotion, Stress and Health Study Section

Meeting Date: 02/19/2019
Council: MAY 2019
Requested Start:
RFA/PA: PA18-345
PCC: 9G-GMH2

Project Title: An anxiety-focused early prenatal intervention for the prevention of common mental disorders in Pakistan
SRG Action: Impact Score:31 Percentile:19
Next Steps: Visit https://grants.nih.gov/grants/next_steps.htm
Human Subjects: 30-Human subjects involved - Certified, no SRG concerns
Animal Subjects: 10-No live vertebrate animals involved for competing appl.
Gender: 1A-Both genders, scientifically acceptable
Minority: 5A-Only foreign subjects, scientifically acceptable
Children: 3A-No children included, scientifically acceptable

Project Year	Direct Costs Requested	Estimated Total Cost
3	437,166	1,626,104
TOTAL	437,166	1,626,104

ADMINISTRATIVE BUDGET NOTE: The budget shown is the requested budget and has not been adjusted to reflect any recommendations made by reviewers. If an award is planned, the costs will be calculated by Institute grants management staff based on the recommendations outlined below in the COMMITTEE BUDGET RECOMMENDATIONS section.

3R01MH111859-03S1 SURKAN, PAMELA

BUDGETARY OVERLAP

RESUME AND SUMMARY OF DISCUSSION: This revision proposes a project to test the biological mechanisms associated with prenatal anxiety, in the context of an ongoing randomized trial of CBT-based anxiety prevention for pregnant women in Pakistan with subthreshold and threshold anxiety disorder, and to add a comparison healthy-control group. This work has the potential to improve the understanding of biological underpinnings of perinatal anxiety and to identify mechanisms associated with this psychological intervention and would be the first examination of inflammatory and endocrine biomarkers in relation to a prenatal psychosocial intervention for anxiety. The PI, co-investigators, and environment are all well suited to complete this work, and the project leverages an ongoing study that increases feasibility, although some overlap in investigator expertise was noted. The panel noted several strengths of the approach, including the repeated measurement of the biological variables, the appropriate design for a mechanistic/mediational model, the addition of a comparison group of healthy pregnant women, and the minimal additional participant burden on top of the ongoing study parameters. The panel also noted several minor to moderate limitations; the most notable of these was the limited support, in the literature and preliminary data, for an association between the inflammatory markers and anxiety in the absence of depression and the relative lack of specificity for the proposed association between anxiety and depression. In addition, the panel indicated that the scope of measures of inflammatory markers was limited and the described plasma collection procedures were unclear and may lead to variability in measurement. The panel was split in their weighing of the relative strengths and limitations of the application, which modestly diminished the impression of the potential high impact of this study.

DESCRIPTION (provided by applicant): While pregnant women frequently suffer from common mental health disorders (CMDs), research on anxiety has been relatively neglected (compared to depression), despite associations with poor pregnancy outcomes and long-term child developmental problems. We previously identified immune and endocrine mechanisms associated with antenatal anxiety, including inflammatory dysfunction and modulation of the progesterone metabolite allopregnanolone (ALLO). Cognitive behavioral therapy (CBT) interventions are highly effective for anxiety and have been shown to affect immune processes, but have not been studied in the context of pregnancy. We now propose leveraging the ongoing randomized evaluation of our CBT anxiety prevention intervention in Pakistan (R01-MH111859) to explore potential biological mechanisms. Our CBT intervention targets both sub-threshold anxiety symptoms and generalized anxiety disorder (GAD) in early- to mid- pregnancy, aiming to both prevent and treat CMDs (GAD and major depressive episodes (MDE)) as well as improve birth outcomes. In this revision application, we propose to additionally study biological correlates of antenatal anxiety (i.e., immune and endocrine functioning) in 300 women: in addition to 200 drawn from our randomized trial (100 intervention, 100 usual care), we will also include 100 healthy women without anxiety or depression. We aim to 1) characterize the "immune phenotype" of anxious women across the peripartum, specifically by measuring the relation among anxiety symptoms and peripheral markers of inflammation within and across women (both anxious and healthy) and between those receiving the intervention and control; 2) determine the relation between levels of ALLO in pregnancy and concurrent anxiety symptoms and future symptoms of postpartum depression (PPD), 3) examine the relation between changes in immune functioning and ALLO levels in anxious pregnancy across time, and 4) examine whether immune function and/or ALLO are mediators or moderators of the association between antenatal anxiety and preterm birth and/or small-for- gestational age. By taking advantage of the ongoing study and routine hospital blood draws, we will minimize participant burden while elucidating the links between perinatal anxiety, correlated biological markers, and the effect(s) of CBT. Perinatal anxiety and its negative impacts on mother and babies, can be ameliorated through greater understanding of its biology.

PUBLIC HEALTH RELEVANCE: Immune and endocrine mechanisms associated with antenatal anxiety, including immune dysregulation modulation of the progesterone metabolite allopregnanolone (ALLO) may be influenced by anxiety prevention interventions, such Cognitive Behavioral Therapy (CBT). We propose leveraging our ongoing CBT anxiety prevention intervention and randomized controlled trial (RCT) in Pakistan (R01-MH111859) to additionally study biological correlates of antenatal anxiety in women with and without anxiety. By taking advantage of the ongoing study and routine hospital blood draws (minimizing participant burden) and creating the first and largest trial to date that connects a perinatal anxiety intervention to its potential biological effects, our study has the potential of helping two vulnerable populations (pregnant women and their infants).

CRITIQUE 1

Significance: 1
Investigator(s): 1
Innovation: 1
Approach: 3
Environment: 1

Overall Impact: The proposed project is a revision (competing continuation) of a prenatal evidence-based CBT intervention (Healthy Mother-Healthy Baby) study currently underway in Pakistan. The goal of the revision is to examine biological (immune and endocrine function) mechanisms in relation to perinatal anxiety in the context of the intervention (N = 200 anxious women, 100 in the intervention and 100 in the enhanced care condition), as well as in healthy controls (N = 100). Because prenatal anxiety has been associated with negative birth and child outcomes, and links have been noted between prenatal anxiety and neuroendocrine and immune measures, this study has high significance and robust scientific rationale. This application provides novel tests of the biological measures in the context of an intervention trial, allowing for assessments of mediator effects of the intervention, and for the relationship between prenatal anxiety and poor birth outcomes. A strong investigative team is paired with well-resourced environments, which further suggests that this study will have high impact.

1. Significance:

Strengths

- Prenatal anxiety has been linked to deleterious outcomes in offspring in both the short and long term and presents as a serious global health issue.
- Identifying relevant biological mechanisms of interventions can inform theory and potentially help modify or target future interventions to improve their effectiveness.
- If this CBT intervention successfully modifies biological risk factors, this intervention might be a cost effective and safer treatment alternative to psychotropic medications.

Weaknesses

- No major weaknesses noted.

2. Investigator(s):

Strengths

- PI Surkan is an Associate Professor with over 180 peer-reviewed publications in the field of maternal and child health (focusing on populations around the globe), including studies that utilize randomized control trials to test interventions.

- Co-I Osborne is the Assistant Director of the Women's Mood Disorders Center and brings expertise in inflammation and immune mechanisms during the perinatal period.
- Co-I Chaudhri is the Head of the Department of Obstetrics and Gynecology of Rawalpindi Medical College and its associated Holy Family Hospital, the source for research participants in this study.
- Other Co-Is provide additional expertise in statistics, hormones, biological sex differences, psychosocial interventions, and global mental health.
- Several members of the team have a history of successful collaboration with one another.

Weaknesses

- No major weaknesses noted.

3. Innovation:

Strengths

- First study to examine immune related biomarkers in relation to anxiety in the context of a prenatal psychosocial intervention.
- Bidirectional feedback between progesterone and immune function highlights the progesterone metabolite allopregnanolone (ALLO) as a potentially important and unexplored biological mechanism of anxiety risk in pregnancy.

Weaknesses

- No major weaknesses noted.

4. Approach:

Strengths

- The attempt to isolate the biological changes resulting from interventions for perinatal anxiety *distinct from perinatal depression* fills an important gap in the treatment literature.
- The repeated measurement of biological variables at four time points across pregnancy and the postpartum period provides a wealth of data points and allows for tests of bidirectional effects over time.
- The study design enables an examination of biological mechanisms that mediate anxiety and birth outcomes.
- There is relatively little added participant burden for the women already taking part in the intervention.
- Focusing on women in Pakistan is a plus, as women in this country have very high rates of stress and pregnancy/delivery complications.
- Low refusal and attrition rates in psychosocial interventions in pregnancy suggest feasibility and the likelihood of successful participant engagement.
- The pilot data support the associations between anxiety and immune related measures.
- The intervention has been developed carefully to conform to local culture and norms, and plans are in place to measure fidelity.

Weaknesses

- Because the intervention has not yet begun, flow rate, recruitment, and retention are still largely unknown.

- ALLO associations in pilot data are underpowered for anxiety. ALLO is associated with depression, but it is unclear if that variable will be relevant in a sample where depression is screened out.
- Literacy is not an inclusion criterion, which means that questionnaires will have to be read to women in those instances, possibly leading to reporting biases.
- Methods such as Compiler Average Causal Effect (CACE) might be useful to consider to control for attrition.

5. Environment:

Strengths

- Johns Hopkins provides excellent resources, including biomarker cores and laboratories, as well as a statistics core to help support this project.
- The Human Development Research Foundation provides the necessary infrastructure for this project in Pakistan.
- The Holy Family Hospital provides space and access to study participants. The OB-Gyn department registers 100 women a day into prenatal care services.
- The proposed study takes advantage of an ongoing treatment protocol, with staff and infrastructure already in place.

Weaknesses

- No major weaknesses noted.

Study Timeline:

Strengths

- The intervention preparation phase is complete.
- The proposed study is designed to piggyback onto the RCT and should not disrupt the original timeline.

Weaknesses

- The timeline is not very detailed. It is unclear as to whether the current proposal needed to provide more information in terms of participant flow, etc., as the RCT was previously described in the original proposal.

Protections for Human Subjects:

Acceptable Risks and/or Adequate Protections

- Risks and protections are well described.

Data and Safety Monitoring Plan (Applicable for Clinical Trials Only):

Acceptable

- Data safety monitoring plans and DSMB are in place and under the charge of NIH.

Inclusion of Women, Minorities and Children:

- Sex/Gender: Distribution justified scientifically
- Race/Ethnicity: Distribution justified scientifically

- For NIH-Defined Phase III trials, Plans for valid design and analysis:
- Inclusion/Exclusion of Children under 18: Excluding ages <18; justified scientifically
- The sample is made up of 300 pregnant women. No direct measures of the children will be undertaken. This study takes place in Pakistan, and all women would fit in the US category of Asian minorities.

Vertebrate Animals:

Not Applicable (No Vertebrate Animals)

Biohazards:

Not Applicable (No Biohazards)

Revision:

- This revision proposes to add a biological mechanism component to the originally funded perinatal intervention study. Progress in the ongoing study suggests a high likelihood of timely and successful completion.

Authentication of Key Biological and/or Chemical Resources:

Not Applicable (No Relevant Resources)

Budget and Period of Support:

Recommend as Requested

CRITIQUE 2

Significance: 3
Investigator(s): 3
Innovation: 3
Approach: 4
Environment: 1

Overall Impact: This is a supplemental application to a funded and ongoing R01 project of a CBT intervention vs. usual care for reducing prenatal anxiety among 1200 pregnant women with sub-threshold anxiety symptoms and generalized anxiety disorder in Pakistan. This supplemental proposal is to add biomarkers of inflammation and endocrine function in 200 women (100 CBT and 100 usual care) from the ongoing study, and 100 additional healthy non-anxious pregnant women. The scientific premise is adequately established and presented, and the overall methodological rigor is noted. However, there are numerous moderate concerns that influenced the overall impact of this proposal, including: ambiguity in anxiety vs. depression in the association with inflammatory state, limited scope of inflammatory marker assessment, inadequate preliminary data, inadequate description of key methods (i.e., plasma collection, laboratory assay quality assurance), and unclear plans for key covariate adjustment.

1. Significance:

Strengths

- The investigation of inflammatory and endocrine markers, in an effort to elucidate the immune and endocrine pathways involved in pregnancy-related anxiety and pregnancy outcomes, can potentially lead to discovering promising therapeutic targets.

Weaknesses

- Implications, or applications for treatment/intervention strategies by testing these particular biomarkers and their functional roles, are unclear in the proposal, leading to the likelihood that the findings are of decreased reproducibility and clinical implications.

2. Investigator(s):

Strengths

- The PI, Dr. Surkan, has assembled a team of investigators whose expertise appears adequate to support these added biological aims to the original project. This proposal builds upon the work of co-investigator Dr. Osborne, whose ongoing K award is on a similar topic.

Weaknesses

- Co-investigator Dr. Mullany's specific and unique role for this subproject is somewhat unclear and appears to somewhat overlap with the role of Dr. Yenokyan.
- There appears to be somewhat of an overlap between roles and expertise of the co-investigators Dr. Osborne and Dr. Klein.

3. Innovation:

Strengths

- The focus of inflammatory correlates of anxiety in pregnancy, independent of depression, is mildly novel in spite of the fact that there is no evidence/likelihood that there are differing inflammatory signatures for anxiety vs. depression.

Weaknesses

- The biomarkers proposed are neither innovative nor comprehensive in investigations of biomarkers of anxiety and depression.

4. Approach:

Strengths

- Leveraging an ongoing study of a large cohort, with clearly defined and elevated anxiety levels at pre- through post-partum, increases the feasibility for this biomarker assessment.
- Assessments of mental health outcomes at multiple time points through pre- and post-partum, as well as pregnancy outcomes in association with biomarker levels, will likely offer insight into pregnancy time-dependent changes that might mirror each other.
- Given the changes in inflammatory activities in normal pregnancy, adding the healthy/non-anxious pregnant women control group will help account for pregnancy-related inflammatory changes.

Weaknesses

- The theorization and clinical implications of studying anxious women, exclusive of depression prepartum to examine postpartum depression (PPD) are unclear, especially in the context of a biological correlates investigation. There aren't likely inflammatory signatures that differentiate

anxiety from depression, or anxiety from anxiety and depression comorbidity. Exclusion of history of prepartum depression, which is shown to be a strong predictor of PPD, may result in misrepresented (i.e., low) rates or severity of PPD, which then may impact the ability to find meaningful associations with proposed biomarkers.

- Related to the comment above, the preliminary data presented are either in pregnant women with mood disorders or increased states of anxiety with unknown levels of depressive mood, which adds to the ambiguity.
- Although strictly building upon what is presented in the preliminary data, the scope of the inflammatory markers assessment is limited. At the same time, functional and theoretical justification for included cytokines/chemokines is lacking, leading to limited knowledge gained in biological pathways that can inform therapeutic/intervention targets.
- Plasma collection procedures are somewhat unclear. It is stated that heparin tubes will be used for blood for immune cell preparation. Is that the case for plasma collection? Blood will be centrifuged for plasma collection up to 8 hours post blood draw. Depending on the condition (e.g., room temp vs. ice), variation in the time between the blood draw and centrifugation will introduce an error due to varying immune activation, protein, or cell degradation, hemolysis, etc.
- It is unclear what key covariates will be included in the statistical models. For example, as weight gain is common, but variable across pregnant women through the perinatal period, and inflammatory markers are highly associated with obesity/adiposity, controlling for BMI at every time point of measurements would be of importance.

5. Environment:

Strengths

- The resources and infrastructure at both Johns Hopkins University and the Human Development Research Foundation in Pakistan, where the study will be conducted, appear adequate for the proposed study. The two entities have an ongoing collaborative relationship, which will be instrumental for the proposed study.

Weaknesses

- No major weaknesses noted.

Study Timeline:

Strengths

- Acceptable as described.

Weaknesses

- No major weaknesses noted.

Protections for Human Subjects:

Acceptable Risks and/or Adequate Protections

- Acceptable.

Data and Safety Monitoring Plan (Applicable for Clinical Trials Only):

Acceptable

- Acceptable.

Inclusion of Women, Minorities and Children:

- Sex/Gender: Distribution justified scientifically
- Race/Ethnicity: Distribution justified scientifically
- For NIH-Defined Phase III trials, Plans for valid design and analysis:
- Inclusion/Exclusion of Children under 18: Excluding ages <18; justified scientifically
- Scientific justification is acceptable.

Vertebrate Animals:
Not Applicable (No Vertebrate Animals)

Biohazards:
Not Applicable (No Biohazards)

Revision:

- This is a revised application to add a sub-study to an ongoing funded R01 as a supplement.

Applications from Foreign Organizations:
Justified

- The proposal to conduct this study in Pakistan is justified.

Resource Sharing Plans:
Unacceptable

- Not provided -- Combined parent and supplement grants > $500,000.

Authentication of Key Biological and/or Chemical Resources:
Unacceptable

- Not provided -- Information on intended assay materials and resources needs to be described.

Budget and Period of Support:
Recommended budget modifications or possible overlap identified:

- It appears that the percent effort for the investigators who are supported by the both original study and this sub-study is unjustifiably high.

CRITIQUE 3

Significance: 1
Investigator(s): 2
Innovation: 2
Approach: 3
Environment: 1

Overall Impact: This is a revision application of an already funded R01 to investigate an intervention in Pakistan with an RCT to randomize 1200 anxious women to cognitive behavioral therapy. The revision proposes to collect biologic data to better understand the biologic mechanisms of anxiety in pregnancy and the effects of CBT on these biologic factors. They propose adding data collection for 300 participants; 200 already participating in the RCT, with 100 receiving CBT intervention and 100 receiving enhanced care, in addition, 100 women will be enrolled as healthy controls with no mood disorders. Additionally, during study blood draw, an additional 20 ml will be collected at enrollment, 2nd trimester, and 3rd trimester, and an additional blood draw at 6 weeks postpartum. Feasibility of adding these subjects and additional assessments was well described, and this additional work appears feasible. This work has potential to be of high impact, given it is elucidating mechanisms of a very common perinatal condition, to better understand treatment, and impact in pregnancy and beyond. It is also a unique opportunity to leverage an already ongoing anxiety prevention intervention trial in the immediate postpartum period, which would be an efficient use of resources and an opportunity to study biologic correlates of perinatal anxiety, and from a clinical RCT specifically, by measuring the association between anxiety and peripheral markers of inflammation. They hypothesize that anxious women will demonstrate a pro-inflammatory state, as compared to non-anxious women, and that this increased inflammatory activity will be reduced by CBT. In addition, the role of allopregnanolone will be examined, given preliminary data showing a relationship. The investigators have a history of funding and publication and with conducting successful funded research in the field. Dr. Rahman is a leading global expert on interventions for perinatal mental health. A team from both Johns Hopkins and Pakistan appears to have the experience needed to conduct this work. Johns Hopkins is very well regarded as a leader in conducting international work in the field and has the appropriate supports needed to successfully complete this work. This work has the potential to improve biologic understanding of perinatal anxiety and inform treatment strategies for a very common perinatal morbidity. The feasibility of completing this revision work in the appropriate timeline seems well justified. Adding a group of healthy controls is a strength of this revision, as it will allow the team to compare biologic processes and inflammation status in a group of anxious women with the intervention, a group of anxious women without the intervention, and compare to healthy controls without anxiety or the intervention. Another strength is the sample size; given the unknown attrition rate, they have increased the attrition rate from 20 to 40%, which is appropriately conservative given the unknown parameters. Minimal score driving limitations include multiple measures of maternal stress, anxiety, and support, and it is not clear from the analysis section how these multiple measures will be accounted for. Also, the inclusion criteria for the determination of anxiety will be made by using the Hospital Anxiety and Depression Scale, although it is not described if this scale has been validated in pregnant/postpartum women. In balance, this revision submission is for highly significant work, by an experienced team already in the field conducting work on this randomized controlled trial requesting additional funds to expand to the collection of inflammatory markers and a control group of women without anxiety, to better understand the biology of anxiety in pregnancy and the impact of treatment with CBT. Minor limitations are balanced by the potential for high impact with an efficient use of funds to expand work already underway.

Protections for Human Subjects:

- No issues

Data and Safety Monitoring Plan (Applicable for Clinical Trials Only):

Acceptable

Inclusion of Women, Minorities and Children:

- Sex/Gender: Distribution justified scientifically

- Race/Ethnicity: Distribution justified scientifically
- For NIH-Defined Phase III trials, Plans for valid design and analysis: Not applicable
- Inclusion/Exclusion of Children under 18: Excluding ages <18; justified scientifically
- No issues identified

Vertebrate Animals:
Not Applicable (No Vertebrate Animals)

Biohazards:
Not Applicable (No Biohazards)

Revision:
- This is an appropriate revision to add biologic sample collection to a currently funded RCT.

Authentication of Key Biological and/or Chemical Resources:
Acceptable

Budget and Period of Support:
Recommend as Requested

THE FOLLOWING SECTIONS WERE PREPARED BY THE SCIENTIFIC REVIEW OFFICER TO SUMMARIZE THE OUTCOME OF DISCUSSIONS OF THE REVIEW COMMITTEE, OR REVIEWERS' WRITTEN CRITIQUES, ON THE FOLLOWING ISSUES:

PROTECTION OF HUMAN SUBJECTS: ACCEPTABLE

INCLUSION OF WOMEN PLAN: ACCEPTABLE

INCLUSION OF MINORITIES PLAN: ACCEPTABLE

INCLUSION OF CHILDREN PLAN: ACCEPTABLE

BUDGETARY OVERLAP: Investigator efforts supported by the both original study and this revision appear high and is not adequately justified.

Footnotes for 3 R01 MH111859-03S1; PI Name: SURKAN, PAMELA J

NIH has modified its policy regarding the receipt of resubmissions (amended applications). See Guide Notice NOT-OD-14-074 at http://grants.nih.gov/grants/guide/notice-files/NOT-OD-14-074.html. The impact/priority score is calculated after discussion of an application by averaging the overall scores (1-9) given by all voting reviewers on the committee and multiplying by 10. The criterion scores are submitted prior to the meeting by the individual

reviewers assigned to an application, and are not discussed specifically at the review meeting or calculated into the overall impact score. Some applications also receive a percentile ranking. For details on the review process, see http://grants.nih.gov/grants/peer_review_process.htm#scoring.

MEETING ROSTER

Biobehavioral Mechanisms of Emotion, Stress and Health Study Section
Biobehavioral and Behavioral Processes Integrated Review Group
CENTER FOR SCIENTIFIC REVIEW
MESH
02/19/2019 - 02/20/2019

Notice of NIH Policy to All Applicants: Meeting rosters are provided for information purposes only. Applicant investigators and institutional officials must not communicate directly with study section members about an application before or after the review. Failure to observe this policy will create a serious breach of integrity in the peer review process, and may lead to actions outlined in NOT-OD-14-073 at https://grants.nih.gov/grants/guide/notice-files/NOT-OD-14-073.html and NOT-OD-15-106 at https://grants.nih.gov/grants/guide/notice-files/NOT-OD-15-106.html, including removal of the application from immediate review.

CHAIRPERSON(S)

BADR, M. SAFWAN, MD
PROFESSOR AND CHAIRMAN
DEPARTMENT OF INTERNAL MEDICINE
DIVISION OF PULMONARY, CRITICAL CARE
AND SLEEP MEDICINE
WAYNE STATE UNIVERSITY
DETROIT, MI 48201

MEMBERS

BIXLER, EDWARD O., PHD *
PROFESSOR
DEPARTMENT OF PSYCHIATRY
PENN STATE MILTON S. HERSHEY MEDICAL CENTER
HERSHEY, PA 17033

BORDERS, ANN E.B., MD *
CLINICAL ASSOCIATE PROFESSOR, PRITZKER SCHOOL OF MEDICINE, UNIVERSITY OF CHICAGO
NORTH SHORE UNIVERSITY HEALTH SYSTEM
DEPARTMENT OF MEDICAL SOCIAL SCIENCES
FEINBERG SCHOOL OF MEDICINE, NORTHWESTERN UNIVERSITY
CHICAGO, IL 60611

BRENNAN, PATRICIA A, PHD *
SAMUEL CANDLER DOBBS PROFESSOR AND CHAIR
DEPARTMENT OF PSYCHOLOGY
EMORY UNIVERSITY
ATLANTA, GA 30322

BRENNER, LISA A, PHD *
DIRECTOR, VA ROCKY MOUNTAIN MIRECC
DEPARTMENTS OF PHYSICAL MEDICINE AND
REHABILITATION, PSYCHIATRY AND NEUROLOGY
SCHOOL OF MEDICINE
UNIVERSITY OF COLORADO
DENVER, CO 80220

BURGESS, HELEN J, PHD
PROFESSOR
DEPARTMENT OF PSYCHIATRY
UNIVERSITY OF MICHIGAN
ANN ARBOR, MI 48109

CHO, JOSHUA HYONG-JIN, PHD, MD *
ASSOCIATE PROFESSOR
COUSINS CENTER FOR PSYCHONEUROIMMUNOLOGY
DEPARTMENT OF PSYCHIATRY AND BIOBEHAVIORAL SCIENCE
DAVID GEFFEN SCHOOL OF MEDICINE AT UCLA
LOS ANGELES, CA 90095

COOK, STEPHANIE HAZEL, DRPH *
ASSISTANT PROFESSOR
DEPARTMENT OF BIOSTATISTICS
DEPARTMENT OF SOCIAL AND BEHAVORIAL SCIENCES
NEW YORK UNIVERSITY
NEW YORK, NY 10003

DE ZAMBOTTI, MASSIMILIANO, PHD *
RESEARCH SCIENTIST
SRI INTERNATIONAL
MENLO PARK, CA 94025

DEVRIES, ANNE COURTNEY, PHD
PROFESSOR
DEPARTMENT OF MEDICINE
SECTION OF HEMATOLOGY AND ONCOLOGY
WEST VIRGINIA UNIVERSITY
MORGANTOWN, WV 26506

DRURY, STACY SCHMIDT, MD, PHD
ASSOCIATE PROFESSOR
DEPARTMENT OF PSYCHIATRY AND BEHAVIORAL SCIENCES
SCHOOL OF MEDICINE
TULANE UNIVERSITY
NEW ORLEANS, LA 70112

FRIEDMAN, ELLIOT MICHAEL, PHD
BERNER HANLEY ASSOCIATE PROFESSOR
DEPARTMENT OF HUMAN DEVELOPMENT
AND FAMILY STUDIES
PURDUE UNIVERSITY
WEST LAFAYETTE, IN 47907

GIANAROS, PETER J, PHD
PROFESSOR
DEPARTMENT OF PSYCHOLOGY
UNIVERSITY OF PITTSBURGH
PITTSBURGH, PA 15260

GRANT, MERIDA M, PHD *
ASSISTANT PROFESSOR
DEPARTMENT OF PSYCHIATRY & BEHAVIORAL NEUROBIOLOGY
THE UNIVERSITY OF ALABAMA AT BIRMINGHAM
BIRMINGHAM, AL 35294

HELLER, WENDY, PHD *
PROFESSOR
DEPARTMENT OF PSYCHOLOGY
UNIVERSITY OF ILLINOIS
CHAMPAIGN, IL 61820

HONG, SUZI, PHD
ASSOCIATE PROFESSOR
DEPARTMENT OF PSYCHIATRY
FAMILY MEDICINE AND PUBLIC HEALTH
UNIVERSITY OF CALIFORNIA, SAN DIEGO
LA JOLLA, CA 92093

KRAUTER, KENNETH S, PHD *
PROFESSOR
DEPARTMENT OF MOLECULAR, CELLULAR AND DEVELOPMENTAL BIOLOGY
COLLEGE OF ARTS AND SCIENCES
UNIVERSITY OF COLORADO
BOULDER, CO 80304

MCCUBBIN, JAMES A, PHD *
PROFESSOR
DEPARTMENT OF PSYCHOLOGY AND PUBLIC HEALTH SCIENCES
CLEMSON UNIVERSITY
CLEMSON, SC 29634

MEAGHER, MARY W., PHD *
PROFESSOR
DEPARTMENT OF PSYCHOLOGICAL AND BRAIN SCIENCES
TEXAS A&M UNIVERSITY
COLLEGE STATION, TX 77843

MURROUGH, JAMES WARREN, MD *
ASSOCIATE PROFESSOR
DEPARTMENT OF PSYCHIATRY
ICAHN SCHOOL OF MEDICINE AT MOUNT SINAI
NEW YORK, NY 10029

NEBLETT, ENRIQUE W JR, PHD
ASSOCIATE PROFESSOR
DEPARTMENT OF PSYCHOLOGY AND NEUROSCIENCE
UNIVERSITY OF NORTH CAROLINA AT CHAPEL HILL
CHAPEL HILL, NC 27599

O'CONNOR, THOMAS G, PHD
PROFESSOR
DEPARTMENT OF PSYCHIATRY
UNIVERSITY OF ROCHESTER MEDICAL CENTER
ROCHESTER, NY 14642

ONG, JASON C, PHD
ASSOCIATE PROFESSOR
DEPARTMENT OF NEUROLOGY
FEINBERG SCHOOL OF MEDICINE
NORTHWESTERN UNIVERSITY
CHICAGO, IL 60611

PACE, THADDEUS W, PHD *
ASSISTANT PROFESSOR
DEPARTMENTS OF PSYCHIATRY AND PSYCHOLOGY
COLLEGES OF NURSING, MEDICINE AND SCIENCE
UNIVERSITY OF ARIZONA
TUCSON, AZ 85721

POWERS, SALLY I, EDD *
PROFESSOR EMERITUS
DEPARTMENT OF PSYCHOLOGICAL AND BRAIN SCIENCES
UNIVERSITY OF MASSACHUSETTS AMHERST
AMHERST, MA 01003

RODRIGUEZ, CARLOS JOSE, MD
PROFESSOR
DEPARTMENT OF EPIDEMIOLOGY AND PREVENTION
DEPARTMENT OF MEDICINE CARDIOLOGY
DIVISION OF PUBLIC HEALTH SCIENCES
WAKE FOREST SCHOOL OF MEDICINE
WINSTON SALEM, NC 27157

SALIM, SAMINA, PHD *
ASSOCIATE PROFESSOR
DEPARTMENT OF PHARMACOLOGICAL AND PHARMACEUTICAL SCIENCES
COLLEGE OF PHARMACY
UNIVERSITY OF HOUSTON
HOUSTON, TX 77204

SCHNYER, DAVID M, PHD *
PROFESSOR
DEPARTMENT OF PSYCHOLOGY
UNIVERSITY OF TEXAS, AUSTIN
AUSTIN, TX 78712

SEPHTON, SANDRA E, PHD *
PROFESSOR
DEPARTMENT OF PSYCHOLOGICAL AND BRAIN SCIENCES
JAMES GRAHAM BROWN CANCER CENTER
UNIVERSITY OF LOUISVILLE
LOUISVILLE, KY 40292

SMOSKI, MORIA J., PHD *
ASSOCIATE PROFESSOR
DEPARTMENT OF PSYCHIATRY AND BEHAVIORAL SCIENCES
DEPARTMENT OF PSYCHOLOGY AND NEUROSCIENCE
DUKE UNIVERSITY
DURHAM, NC 27710

STARKWEATHER, ANGELA RENEE, PHD
PROFESSOR AND ASSOCIATE DEAN FOR ACADEMIC AFFAIRS
CENTER FOR ACCELERATING PRECISION PAIN SELF-MANAGEMENT
SCHOOL OF NURSING
UNIVERSITY OF CONNECTICUT
STORRS, CT 06269

STREETER, CHRIS CONWAY, MD *
ASSOCIATE PROFESSOR
BOSTON YOGA RESEARCH CENTER
DEPARTMENT OF PSYCHIATRY AND NEUROLOGY
SCHOOL OF MEDICINE
BOSTON UNIVERSITY
BOSTON, MA 02114

SU, SHAOYONG, PHD *
ASSOCIATE PROFESSOR
GEORGIA PREVENTION INSTITUTE
MEDICAL COLLEGE OF GEORGIA
AUGUSTA UNIVERSITY
AUGUSTA, GA 30912

WEIERICH, MARIANN R, PHD *
ASSOCIATE PROFESSOR
DEPARTMENT OF PSYCHOLOGY
HUNTER COLLEGE
NEW YORK, NY 10065

WONG, MARIA M., PHD
PROFESSOR AND DIRECTOR, DEVELOPMENT AND RESILIENCE LAB
DEPARTMENT OF PSYCHOLOGY
IDAHO STATE UNIVERSITY
POCATELLO, ID 83209

YOUNGSTEDT, SHAWN D, PHD
PROFESSOR
COLLEGE OF NURSING AND HEALTH INNOVATION
COLLEGE OF HEALTH SOLUTIONS
ARIZONA STATE UNIVERSITY
PHOENIX, AZ 85004

SCIENTIFIC REVIEW OFFICER

SHAPERO, BENJAMIN GREENBERG, PHD
SCIENTIFIC REVIEW OFFICER
CENTER FOR SCIENTIFIC REVIEW
NATIONAL INSTITTUES OF HEALTH
BETHESDA, MD 20892

EXTRAMURAL SUPPORT ASSISTANT

SMITH, JACQUELINE ELAINE
EXTRAMURAL SUPPORT ASSISTANT
CENTER FOR SCIENTIFIC REVIEW
NATIONAL INSTITUTES OF HEALTH
BETHESDA, MD 20892

* Temporary Member. For grant applications, temporary members may participate in the entire meeting or may review only selected applications as needed.

Consultants are required to absent themselves from the room during the review of any application if their presence would constitute or appear to constitute a conflict of interest.
